# Understanding why EmpaTeach did not reduce teachers’ use of violence in Nyarugusu Refugee Camp: A quantitative process evaluation of a school-based violence prevention intervention

**DOI:** 10.1371/journal.pgph.0001404

**Published:** 2023-06-14

**Authors:** Camilla Fabbri, Timothy Powell-Jackson, Katherine Rodrigues, Alexandra De Filippo, Michael Kaemingk, Gerard Torrats-Espinosa, Baptiste Leurent, Elizabeth Shayo, Vivien Barongo, Karen M. Devries

**Affiliations:** 1 London School of Hygiene and Tropical Medicine, London, United Kingdom; 2 International Rescue Committee, New York, New York, United States of America; 3 Behavioral Insights Team, Brooklyn, New York, United States of America; 4 Columbia University, New York, New York, United States of America; 5 University College London, London, United Kingdom; 6 National Institute for Medical Research, Dar es Salaam, Tanzania; University at Buffalo, UNITED STATES

## Abstract

EmpaTeach was the first intervention to address teacher violence to be tested in a humanitarian setting and the first to focus on reducing impulsive use of violence, but a cluster randomised trial found no evidence that the intervention was effective in reducing physical and emotional violence from teachers. We aimed to understand why. We conducted a quantitative process evaluation to describe the intervention implementation process (what was implemented and how); examine teachers’ adoption of positive teaching practices (was the content of the intervention taken up by participants), and test mechanisms of impact underlying the program theory (how the intervention was supposed to produce change). Despite participation in the intervention activities and adoption of intervention-recommended strategies (classroom management and positive disciplinary methods), we show that teachers who used more positive discipline did not appear to use less violence; and teachers in intervention schools did not experience gains in intermediate outcomes such as empathy, growth mindset, self-efficacy or social support. Our findings suggest that the intervention did not work due to the failure of some key hypothesised mechanisms, rather than because of implementation challenges.

## Introduction

### Background

The detrimental effects of violence on children’s physical and mental health as well as on a wide range of socio-economic outcomes are well known [1–3]. After decades of research into the causes and consequences of violence against children, academics, practitioners and donors have turned their focus to understanding how to effectively prevent violence in and through schools [4, 5]. School is meant to be a safe and protective environment where children can focus on building knowledge, skills and competencies that will allow them to achieve positive educational, economic, and social outcomes in adulthood. However, school is also a setting where children experience violence from both teachers and peers [6].

In resource-constrained settings teacher violence often takes the form of corporal punishment used to manage overcrowded classrooms and discipline misbehaving students. The use of violent discipline and corporal punishment by teachers may be particularly widespread in settings characterised by large class sizes, unqualified school staff, weakened social ties, and norms that condone it, even when violence is formally prohibited [7–9]. According to a recent systematic review of 53 studies, lifetime prevalence of school corporal punishment was above 70% in Africa and Central America, and past-week prevalence was above 40% in Africa and Southeast Asia. In primary schools these estimates were higher with lifetime prevalence above 80% and past-week prevalence around 50% in some settings [10]. Official statistics from humanitarian settings are scant, however evidence suggests that the risk of violence to displaced and refugee children may be even higher due to heightened stressors on education service provision and individual teachers [11–13]. Humanitarian crises and displacement are likely to expose individuals and communities to economic strain, hunger, as well as to stress and traumatic events [14, 15]. Additionally, exposure to violence before, during or after displacement is likely to be compounded by traditional social norms that condone or promote use of violence leading to the normalisation of violence [16].

To date only a handful of interventions to prevent teacher violence have been rigorously tested globally [17–21] and the evidence on what works and what doesn’t is still limited, especially in refugee and emergency settings [22]. Among 93 programmes aimed at preventing forms of violence against children in and through schools in the Global South, only 7% targeted teachers’ use of corporal punishment Of these programmes, 23% targeted multiple forms of violence, 17% targeted gender-based violence, 23% targeted bullying, 14% targeted sexual violence, 8% targeted aggression and 8% targeted other forms of violence against children and few studies reported the results of evaluations assessing the programme implementation and mechanisms [5]. Little is known about what the essential intervention components and key mechanisms of impact of successful school-based violence prevention interventions are. This lack of evidence precludes practitioners’ and policy makers’ ability to develop and/or adapt effective programmes to prevent and respond to violence in schools. When interventions fail to produce the expected results, it is even more important to conduct complementary process analyses to unpack where, how, and why breakdowns of the intervention theory may have happened to inform future efforts.

In this study, we conducted a quantitative process evaluation of the EmpaTeach intervention to investigate why the intervention was not successful in lowering levels of teacher violence in schools in Nyarugusu Refugee Camp, Tanzania. EmpaTeach was, to the best of our knowledge, the first school-based violence prevention intervention to be rigorously evaluated in a humanitarian setting. Between January and March 2019 and, the International Rescue Committee (IRC), implemented the 10-week teacher-led intervention, aimed at preventing violent discipline in primary and secondary schools in Nyarugusu Refugee Camp in Tanzania. The intervention was designed to address high levels of school violence known to exist in the camp, despite such practices being explicitly prohibited. A cluster randomised controlled trial of the intervention found no evidence of an effect of EmpaTeach on physical violence from teachers to students (primary outcome) and no measurable effect on students’ experiences of emotional violence, depression symptoms and school attendance (secondary outcomes) [23]. However, post-hoc analyses of the trial data suggested that the intervention improved some intermediate outcomes: teachers in intervention schools used more positive discipline strategies, showed attitudes that were less supportive of violent discipline and reported improved self-regulation compared to those in control schools [23].

The null effects on primary and secondary outcomes could be attributed to the magnitude of change in intermediate outcomes that may have been too small to lead to changes in teacher behaviour; to the possibility that teachers may have used new disciplinary and classroom management strategies, but they did so in addition, rather than in replacement, of traditional corporal punishment approaches; or to the absence of a sufficient causal link between these intermediate outcomes and teachers’ use of violence. The process evaluation that we present here relies on various evaluation frameworks [24–26] and data sources to a) describe the intervention implementation process (what was implemented and how); b) examine teachers’ adoption of positive teaching practices (was the content of the intervention taken up by participants), and c) test mechanisms of impact underlying the programme theory (how the intervention was supposed to produce change).

### Intervention package

EmpaTeach is a 10-week low-intensity, peer-led intervention targeted at teachers. Its aim is to reduce and prevent teachers’ use of violence in the classroom, as well as improve teacher wellbeing. The intervention, which was co-designed with refugee teachers by the International Rescue Committee in partnership with the Behavioral Insights Team, draws on behavioural insights and cognitive-behavioural therapy to upskill teachers with positive classroom management, alternative discipline strategies, and self-regulation and wellbeing techniques. The programme uses a booklet to guide teachers through 12 in-person sessions during which they engage in interactive exercises to identify personal triggers, explore how their thoughts and feelings lead to reactions, and learn and plan when to use new strategies to manage classroom dynamics and respond to student misbehaviours. Teachers in intervention schools are grouped with peers who serve as their support network and new social reference point; groups are led by one of the members, after they receive a short facilitation training.

The intervention instructs teachers on best practices in education (e.g., providing feedback, encouraging growth mindsets, checking for understanding, using questioning techniques, etc.) and classroom management to improve their ability to effectively deal with large overcrowded classrooms and maintain student focus. Strategies to manage the classroom include setting a lesson objective, creating classroom rules, using discipline in a consistent manner, using the classroom space, and using positive discipline. In the positive discipline session teachers are exposed to a number of encouragement (e.g., cheering, praising etc.), redirection (e.g. moving in the classroom, changing tone of voice, clapping etc.) and reflection techniques that they can use to provide emotional support to well-performing students, to de-escalate misbehaviours, and to punish those who engage in disruptive behaviours.

### Programme theory of the EmpaTeach intervention

The EmpaTeach intervention rests on the premise that overcrowding and resource constraints in humanitarian settings make classroom management challenging and generate stress for teachers. In the absence of alternative discipline tools, it was hypothesized that teachers resort impulsively to the use of violent discipline to punish students’ behaviours that are perceived as problematic.

In-person weekly sessions were designed to provide teachers with an opportunity to reflect on their own experiences, learn and practice new classroom management and positive disciplinary methods with peers, while practising self-regulation and wellbeing techniques. Group dynamics and interactions during these meetings were considered important to prompt teachers to reflect on current practice, role-play new strategies and foster the creation of new social norms and attitudes towards corporal punishment. The intervention content (i) asked teachers to practice self-affirmation to reduce self-threat, encourage openness to change, and build self-efficacy [27]; (ii) prompted teachers to take different perspectives and to support each other and their students to build empathy [28]; and (iii) emphasised teachers’ potential to change and overcome challenges with effort [29]. The intervention theory was primarily based on the idea that teachers’ adoption of positive classroom management and alternative discipline strategies would lead to the replacement of violence in favour of newly acquired strategies. In other words, teachers would test and use the intervention-recommended strategies to find them so effective that they would resort less to the use of violent discipline. Additionally, improved teacher skills and competencies would contribute to improvements in attitudes towards violence and self-control which in turn would lead to a reduction in teachers’ use of violence.

Homework assigned to teachers on a weekly basis was designed to induce a process of behavioural activation through repeated practice of new discipline and classroom management strategies both in class and outside of school hours. Finally, the social support and feedback opportunities provided by the peer group and by a named supporter were supposed to encourage teachers throughout the change process and reinforce new norms and practices by providing a new reference group.

The intervention focused on building new skills for teachers and intentionally avoided discouraging the use of violence in the classroom because addressing attitudes on violence explicitly would have required expert facilitation that was not available in the camp and would have reduced the scalability of the intervention [23]. It was hypothesised that by practicing the new strategies teachers would be persuaded to abandon the use of corporal punishment in favour of the new, more effective positive disciplinary methods (replacement effect), and that empathy building exercises coupled with social reinforcement provided by the peer groups would promote a change in self-regulation and attitudes towards violent discipline.

## Methods

### Ethics statement

The Preventing Violence Against Children in Schools (PVACS) study was approved by the London School of Hygiene & Tropical Medicine Ethics Committee (ref. 16000) and the Tanzania National Institute for Medical Research (ref. NIMR/HQ/R.8a/Vol.IX/2920).

The following consent procedures were followed for the cross-sectional surveys [23]: headteachers provided consent for data collection and intervention implementation in schools, and consent to approach individual students; students provided informed assent, and individual teachers provided informed consent. In consultation with camp stakeholders, it was deemed appropriate in this setting to seek headteachers’ consent for children’s participation as they had full responsibility for students during school hours and seeking active parental consent would have precluded participation of many children, given high numbers of unaccompanied youth in the camp. Teachers and headteachers provided verbal consent to IRC’s classroom observations which were conducted as part of the monitoring of the intervention implementation. Teacher attendance and homework records were collected as part of teachers’ participation in the EmpaTeach intervention.

### Study setting

The PVACS study included a cluster randomised controlled trial to evaluate the effectiveness of the EmpaTeach intervention to reduce violence from teachers to students in primary and secondary schools in Nyarugusu Refugee Camp in Tanzania. At the time of the study Nyarugusu was hosting around 150,000 refugees from the Democratic Republic of Congo and Burundi, some of whom had been there since the mid-1990s when the camp was established. The camp is operated by the United Nations High Commission for Refugees and the Tanzania Ministry of Home Affairs. The IRC provides education and gender-based violence response services and, at the time of the study, all child protection services. The camp is largely divided between Congolese and Burundian populations who reside in different zones; schools follow the curriculum of the country of origin. According to a needs assessment conducted in late 2017 more than 2 in 3 students reported feeling safe in school; however several child protection concerns were identified related to poor facilities, overcrowded classrooms, and the risk of violence in and on the way to and from school [30]. Formative research conducted prior to the PVACS trial also highlighted that resource constraints and overcrowding were important stressors for teachers in the camp. In this context, EmpaTeach was designed to improve teacher wellbeing and reduce stress-induced violence in schools [7].

### Evaluation approach

We designed the process evaluation prospectively in September 2018 in collaboration with the International Rescue Committee and Innovations for Poverty Action. The original analytical plan was designed to focus on mediation analysis of teachers’ and students’ outcomes and less on intervention implementation. In light of our trial findings, in this study we extended our approach to follow each step in the hypothesised pathways that link intervention and outcomes, starting with reach and adoption and moving on to mechanisms of action of the intervention. Reach refers to the extent to which the intended audience comes into contact with the intervention [31] while adoption is defined as the intentional decision, in our case by teachers, to employ an innovation or practice; adoption is also referred to as “uptake” [32]. To measure reach, we used teachers’ attendance records to explore whether teachers actually participated in the programme. To measure adoption, we used a combination of teacher-classroom observations and survey data to examine whether teachers implemented the intervention-recommended strategies.

Additionally, we explored the main pathways through which the intervention was supposed to produce change (*mechanisms*): a) we tested for the presence of a replacement effect by looking for an association between teachers’ use of intervention-recommended strategies and their use of violence and b) estimated the intervention effect on teachers’ empathy, growth mindset, self-efficacy and social support which were all hypothesised to complement and sustain the use of the EmpaTeach strategies and to promote changes in self-regulation and attitudes towards violence.

### Data

Our analyses drew on various sources of data (Table 1). We used (1) teacher attendance and homework completion records compiled by EmpaTeach group coordinators at the beginning of each intervention session and delivered to IRC staff on a weekly basis; (2) data from unannounced teacher classroom observations (TCOs) collected by the IRC’s programme team using standardised checklists as part of their monitoring activities; (3) and two rounds of cross-sectional surveys with teachers and students conducted within the school setting by the research team as part of the cluster randomised-controlled trial before (baseline) and 2 months after the end of the intervention (midline). All datasets used for analyses contained anonymised data and only participant IDs were used to link datasets.

**Table 1:** Description of data sources.

Data source	Sample	Measures included
Teacher attendance and homework records (Jan-March 2019)	600 teachers in 14 intervention schools^	Individual teacher attendance and homework completion records compiled by group coordinators at each intervention session.
Teacher classroom observations (Feb-March 2019)	Random sample of 153 teachers (103 in intervention schools and 50 in control schools) in 14 intervention schools and 9 control schools. Each teacher was observed by two raters who scored independently, average scores were used in analyses	Observation of teacher behaviour in classroom assessed using a 22-items checklist measuring four main constructs: time on task (5 items), teaching practices (7 items), classroom management (5 items), and emotional support (5 items). Scoring was done on a Likert scale: 1 = no evidence/negative, 2 = tried but poorly, 3 = good effort, 4 = perfect. An additional checklist was used to count the number of encouragement and positive discipline strategies used by teachers.
Teacher cross-sectional surveys (November 2018, May 2019)	Random sample of 488 teachers at baseline and 510 teachers at midline in 27 schools (both intervention and control)	Surveys collected information on demographics, experiences of the school environment, attitudes towards violence, use of violence, skills and competencies, and mental health. Teachers’ self-reported use of violence as was calculated as a binary variable that took a positive value if the respondent reported using any act of physical violence in the past week against students using questions adapted from the ISPCAN Child Abuse Screening Tool–Child Institutional (ICAST-CI) [34]. Teachers’ empathy was calculated as a 0–60 score using the Barrett-Lennard Relationship Inventory [35]. Teachers’ self-efficacy was calculated based on three items adapted from the self-efficacy scale [36] scored on a 1–4 Likert scale. Teachers’ social support was measured using two items “Do you feel that there is anybody at your school you can talk to if you feel unhappy about work?” and “Thinking about your school as a whole, do you feel like you are part of a team?” scored on a 1–4 Likert scale. Growth mindset was calculated based on five items adapted from a growth mindset scale [29] scored on a 1–4 Likert scale. Teachers were asked multiple choice questions about their classroom strategies under four scenarios and answers were classified as positive or negative strategies according to whether they were in line with the intervention content. The four scenario questions asked about how teachers would react when: a) they would get frustrated in the classroom and would need to calm themselves down, b) students would be found chatting during class therefore disturbing the lesson, c) students would arrive late for class, d) a student would be performing well in class giving the correct answer to a question/exercise*.
Student cross-sectional surveys (November 2018, May 2019)	Random sample of 1,493 students at baseline and 1,619 students at midline in 27 schools	Surveys collected information on demographics, experiences of the school environment, attitudes towards violence, experiences of violence, skills and competencies, and mental health. Students’ self-reported experiences of violence from school staff was calculated as a binary variable that took a positive value if the respondent reported experiencing any act of physical violence in the past week using questions adapted from the ISPCAN Child Abuse Screening Tool–Child Institutional (ICAST-CI) [34].

Notes:

^ Analyses were conducted on 597 teachers for whom attendance data was available.

* Detail on the classification of strategiescan be found in S1 Text.

Routine monitoring data such as teacher attendance and homework records were collected from IRC’s programme team and were not validated and were missing values for 3 teachers. The scales included in the teacher classroom observations checklist were pre-tested as part of prototyping and piloting before the implementation of the intervention by IRC staff and inter-rater reliability and validity of the indicators have been assessed elsewhere [33]. All measures included in the cross-sectional surveys were cognitively tested and adapted to the local context.

### Analysis

First, we present descriptive statistics for indicators of teachers’ participation in intervention activities. Reach was measured using two indicators: teachers’ individual attendance to group sessions and teachers’ homework completion.

Then, we used an intent-to-treat approach to assess differences in teachers’ uptake of positive classroom management and alternative disciplinary strategies between intervention and control schools, drawing from teacher classroom observations data and PVACS survey data. Adoption was measured as the number and quality of classroom management and positive disciplinary practices that teachers were observed implementing and self-reported using in school. We used t-tests adjusted by clustering to assess the difference in means for continuous variables, such as the quality (score) of how teachers applied the different types of practices and the number of practices they were observed using in the classroom.

Finally, we used multilevel logistic regression models using teacher reported survey data at midline to test the mechanisms to impact of the intervention. To assess the association between teachers’ use of intervention-recommended strategies and their use of violence in the classroom we used pooled data from intervention and control schools to implement four separate models where teachers’ use of violence was included as the outcome and the number of practices they reported using in each of four classroom scenarios as the main exposure, controlling for teacher covariates. We checked the robustness of our findings by replicating the same analyses using student-reported experiences of violence from teachers as the outcome (in line with our main trial analyses) and a school-level mean of teachers’ use of the practices as the main exposure, controlling for student covariates. All models accounted for clustering at the school level and controlled for intervention assignment. Then we used an intent-to-treat approach to test whether the intervention led to changes in teachers’ empathy, growth mindset, self-efficacy, and social support which were hypothesised to be potential mediators (since a variable can only be a mediator of the intervention effect if there is a significant effect of the intervention on it) of the intervention effect on attitudes towards violence and self-control. All data were analysed in Stata/SE 17, and considered statistically significant at the two-sided 5% significance level.

## Results

### Reach: Teachers’ participation in intervention activities

The intervention was implemented in all 14 schools that were randomly assigned to receive the intervention. Overall, 600 teachers formed 77 peer-led EmpaTeach groups of varying sizes (each group included between 3 and 15 teachers (although the average group was intended to include 7–10 teachers). Attendance records were available for 597 teachers. Table 2 offers an overview of teachers’ participation in the intervention activities. Overall, around half of the teachers (52.4%) attended at least 10 out of 12 group sessions. Ten teachers did not attend any of the EmpaTeach sessions (four of which transferred to intervention schools during the intervention implementation period). Few teachers completed assigned homework: 24% of teachers did not complete any of the homework assignments and fewer than 1 in 3 teachers completed 80% or more of the homework practice. Homework completion was higher in secondary schools while attendance did not vary by level of schooling.

**Table 2:** Indicators of teachers’ participation in intervention activities.

Attendance to EmpaTeach weekly sessions	n/N or mean	% (SD)
Attendance at individual level		
Low: 0–5 session	99/597	16.6%
Medium: 6–9 sessions	185/597	31.0%
High: 10–12 session	313/597	52.4%
**Completion of homework assignments**		
Homework completion at individual level		
0–3 assignments	294/597	49.2%
4–6 assignments	131/597	21.9%
7–8 of assignments	172/597	28.8%

Notes: Homework records collected by the IRC for the first four sessions of EmpaTeach were missing, therefore homework completion is calculated over a total number of 8 completed assignments.

As previously reported [23], the intervention implementation was affected by a major incident whereby 20% of teachers in the camp were laid off from their jobs during the intervention delivery period (January 2019). Although most teachers were subsequently re-hired in the same role within a period of 2 weeks, EmpaTeach intervention groups were disrupted over a period of approximately 4 weeks.

### Adoption: Teachers’ use of intervention-recommended strategies

**Fig 1:**
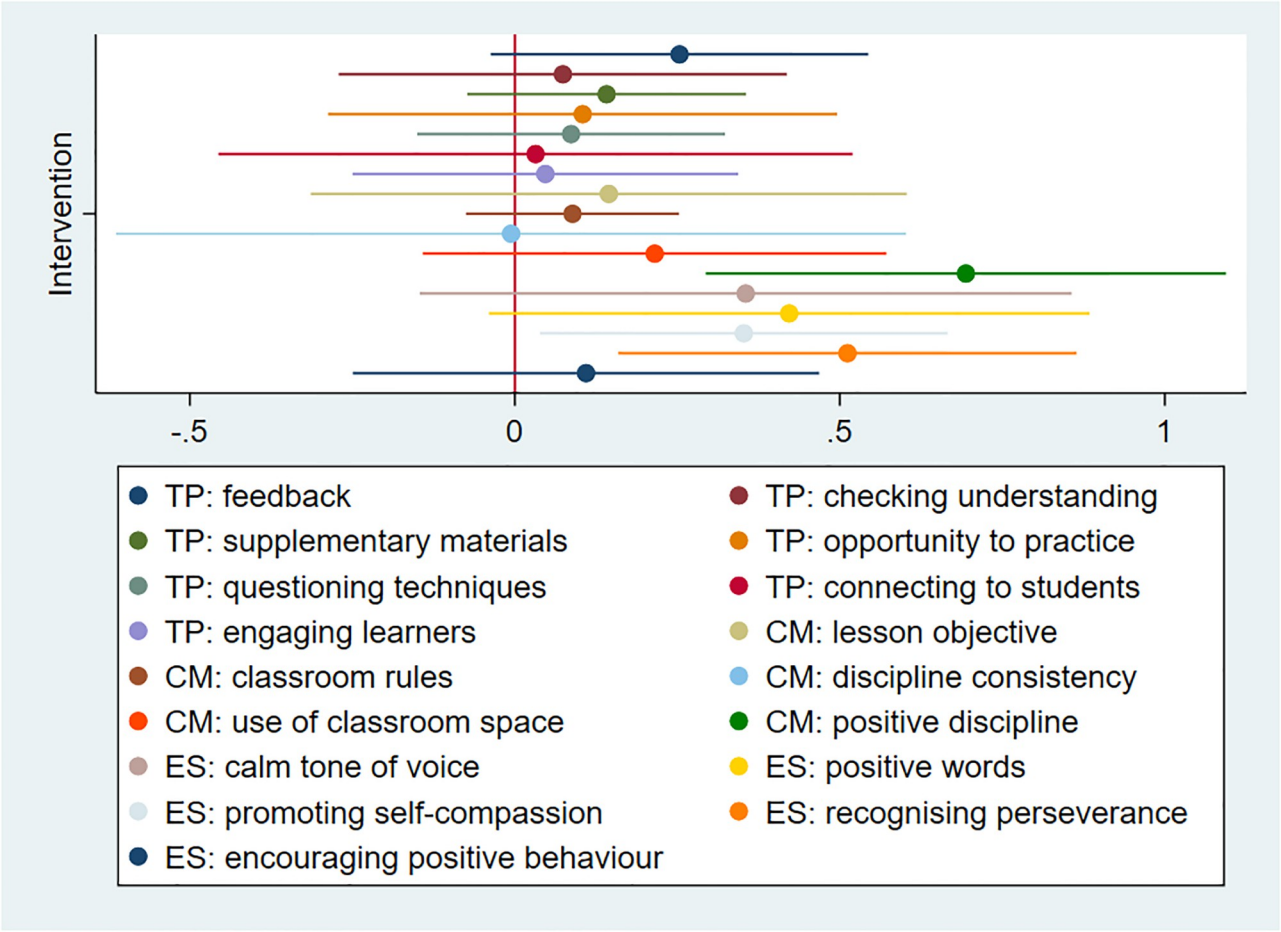
Effect of intervention on teachers’ use of positive practices.

To explore teachers’ use of the strategies that they learnt during intervention sessions we relied on data from observation of teachers’ behaviours in the classroom in both intervention and control schools. Data offered insights into teacher’s use of teaching practices (TP) and classroom management strategies (CM), and their provision of emotional support to students (ES). There were generally no differences between teachers in intervention and control schools in the use of recommended teaching practices and classroom management strategies; however, teachers in intervention schools were significantly better at using positive disciplinary practices than those in control schools (mean score = CM: positive discipline was 1.488, SD = 1.010 in control schools and 2.182, SD = 1.382 in intervention schools, p = 0.002) and at providing emotional support to students by promoting self-compassion (mean = 1.494, SD = 0.904 in control schools and mean = 1.846, SD = 0.894 in intervention schools, p = 0.029) and recognising perseverance (mean = 1.434, SD = 0.853 and mean = 1.946, SD = 1.269 in control and intervention schools respectively, p = 0.006) (Fig 1). In Table 3 we examined more closely how frequently teachers used specific emotional support methods and positive discipline. We found that teachers in intervention schools used significantly more cheering than teachers in control schools, but generally no other statistically significant differences were observed in use of encouragement strategies. We note however that these differences were quite large (use of praise doubled in intervention schools) and our analyses may have been underpowered to detect them. On the contrary, teachers in intervention schools were observed using a statistically significant higher number of alternative disciplinary practices such as redirection techniques (such as moving closer to students or changing students’ seats) to manage student misbehaviours and disturbances in the classroom (Table 3). These results were broadly in line with findings from the intent to treat trial analyses (already presented in another publication and replicated here using mixed models) conducted using teacher self-reported use of these strategies to a series of scenario questions in the PVACS surveys (S1 Table).

**Table 3:** Number of specific encouragement and positive discipline techniques used by teachers.

	Control (N = 50)	Intervention (N = 103)	t-test
	Mean (SD)		P
**Encouraging positive behaviour**			
Cheering *When a student does something right*, *the teacher has other students clap for him or her*.	1.652	3.108	0.077
	(4.794)	(4.410)	
Praise *Praise students for good behavior and effort so they are encouraged to repeat these good actions and learn to work hard*.	0.818	1.537	0.224
	(2.453)	(4.799)	
Additional activities *Some students excel in class and this means that they understand class materials well and are ready to learn more*. *One way to promote engagement is to give them additional activities related to a topic of interest*.	0.000	0.049	0.235
	(0.000)	(0.408)	
Student of week *When students are consistently well-behaved for a week or more*, *recognize their efforts and good behavior in front of the class*. *Every Friday*, *choose a small number of students to be “Students of the Week”*.	0.000	0.000	N/A
	(0.000)	(0.000)	
**Positive discipline**			
Move close *When a student misbehaves*, *the teacher moves closer to the student and looks at him or her*.	0.338	0.988	0.033
	(1.364)	(2.212)	
Lower voice *Sometimes speaking really softly can be more powerful than speaking loudly*. *Change the volume of the voice to regain students’ attention*.	0.000	0.117	0.089
	(0.000)	(0.672)	
Sudden silence *As soon as a student misbehaves*, *the teacher stops talking and looks directly at the misbehaving student*.	0.080	0.437	0.073
	(0.594)	(1.756)	
Move around class *The teacher moves around the classroom while teaching so students are engaged with the lesson and behave better*	4.661	4.243	0.481
	(3.606)	(3.197)	
Polite pose *When a student is distracted or misbehaving*, *the teacher points at the student and says “take a polite pose”*. *“Polite Pose” means to sit in your seat with you back straight and arms folded in front of you*.	0.278	0.718	0.048
	(1.139)	(1.447)	
Clapping *When a group of students are misbehaving or unsettled*, *the teacher can quickly and loudly clap their hands together two or three times*. *The teacher can instruct the students to clap back the same number of times and then sit quietly when they hear the teacher clap*.	0.008	0.311	0.110
	(0.061)	(1.866)	
Change seats *If students are being disruptive by talking to each other in class*, *move one of the students to sit in another location in the classroom*.	0.130	0.451	0.039
	(0.601)	(1.240)	

Notes: Encouragement techniques were part of the “Encouraging Positive Behaviour” practices under Emotional Support (ES) and Redirection Techniques were part of the “Positive discipline” practices under Classroom Management (CM). The table shows the mean number of techniques employed by teachers observed in intervention and control schools. Standard deviations are clustered at the school level.

The figure shows the regression coefficients and 95% confidence interval for the effect of the intervention on the quality assessment of recommended practices (score 1 to 4, where higher values correspond to better use of each practise). TP = Teaching Practices, CM = Classroom Management practices, ES = Emotional Support.

### Mechanisms of impact

#### Replacement of violence with positive strategies to manage and discipline students

Using teacher self-reports, we found that the hypothesised replacement of violence with positive discipline strategies probably did not take place: Table 4 shows that none of the positive intervention practices were associated with significant decreases in use of physical violence from teachers. We replicated the same analyses using student self-reported experiences of violence and school-level indicators of teachers’ use of positive practices as exposure (students cannot be linked to individual teachers, since some students have multiple teachers) and results are reported in S2 Table. We found no evidence that teachers’ use of recommended EmpaTeach strategies was associated with lower use of violent discipline. If anything, teachers’ use of praise strategies was associated with higher odds of students reporting experiences of physical violence.

**Table 4:** Association between use of positive strategies and teachers ‘self-reported use of violence at midline.

	Physical violence in past week	
Teachers’ self-reported use of classroom management and discipline strategies	Unadjusted OR (95%CI)	Adjusted OR (95%CI)
Number of strategies to manage stress in class	0.96	0.97
	(0.74–1.26)	(0.73–1.28)
Number of strategies to manage students disturbing in class	0.98	0.96
	(0.79–1.22)	(0.77–1.20)
Number of strategies to manage students arriving late to class	0.70	0.65
	(0.37–1.33)	(0.33–1.26)
Number of strategies to praise students	1.11	1.10
	(0.90–1.37)	(0.89–1.37)

Notes: The table displays odds ratios (95% CI). N = 510 teachers interviewed at midline in all 27 schools. Adjusted analyses include teacher sex, age, country of origin, education level, meals eaten yesterday, number of children, position within the school and days of absence in past month. Both models control for treatment status.

#### Teachers’ socio-emotional skills: Empathy, self-efficacy and social support

Table 5 shows that the intervention did not significantly impact teachers’ empathy levels, growth mindset, self-efficacy in the classroom or perceived social support; there was no statistically significant difference in these outcomes between teachers in control and intervention schools. These factors could not therefore mediate the effects on teachers’ attitudes and self-control as hypothesised in the programme theory.

**Table 5:** Intervention effect on teachers’ empathy, perspective taking and social support at midline.

	Control	Intervention	Difference	
	Mean (SD)		Marginal effect (95% CI)	P
Empathy towards students (0–60)	42.6 (6.85)	42.7 (6.99)	-0.04 (-1.33–1.25)	0.954
Growth mindset (1–4)	2.90 (0.37)	2.93 (0.34)	0.03 (-0.05–0.11)	0.435
Self-efficacy (1–4)	3.29 (0.44)	3.29 (0.45)	0.01 (-0.09–0.10)	0.905
Social support (1–4)	2.90 (0.72)	2.99 (0.70)	0.08 (-0.04–0.21)	0.167

Notes: Marginal effects and confidence intervals are based on mixed linear models. All models are adjusted for randomisation strata (school nationality and level).

## Discussion

### Summary of main findings

Our results showed that attendance and participation in the intervention were overall adequate considering the challenging context in which EmpaTeach was implemented and the fact that implementation was quite severely disrupted. We also demonstrated that teachers learnt and used new classroom management strategies and positive disciplinary methods confirming that the content of the intervention was accessible and relevant for participants. However, teachers’ adoption of the EmpaTeach strategies was not associated with their use of violence: teachers who used more positive discipline did not appear to use less violence; and teachers in intervention schools did not experience gains in empathy, growth mindset, self-efficacy or social support. This suggests that the hypothesized replacement of violence with nonviolent alternative discipline did not occur, and that intervention activities did not have an appreciable effect on intermediate outcomes thought to contribute to impulsive use of violence.

### Understanding why EmpaTeach did not work

Although our results suggest that what *did not work* in EmpaTeach was related to the design of the intervention and not to the context in which it was implemented, we cannot exclude that the same intervention implemented in a non-refugee environment may have yielded different results.

Adoption of intervention-recommended alternative discipline techniques presented in the EmpaTeach booklet such as moving closer to or changing seats to misbehaving students or using a sudden silence to regain attention in the classroom, was significantly higher in intervention versus control schools. However, we found no significant difference between intervention and control schools in teachers’ use of encouragement strategies that were designed to reward positive behaviours of students, aside from cheering. It is possible that these encouragement techniques were the easiest to be adopted (and in terms of frequency, teachers in all schools reported using them more frequently than other strategies) and therefore participation to EmpaTeach sessions was not necessary to enable teachers’ uptake.

The intervention theory was based on the idea that teachers would test and practise these new strategies and that they would ultimately find them so useful and effective in managing student misbehaviour that teachers would substitute the use of violent discipline with these newly acquired methods. The intervention therefore did not explicitly discourage use of corporal punishment and relied on practice of new strategies, coupled with social support, to lead to changes in violent behaviour. Since in the main trial analyses we observed no measurable difference in the use of violence between intervention and control schools, we tested the validity of this theory and found that teachers’ adoption of the EmpaTeach strategies was not associated with their use of violence: teachers who used more positive discipline did not appear to use less violence. It is possible that teachers simply expanded the range of methods they used to manage student misbehaviours and that the replacement of violent behaviours could not take place without formal discouragement of corporal punishment. In contexts like Nyarugusu Refugee Camp where, despite formal bans, corporal punishment is condoned and perceived as a necessary and useful approach to discipline, it may be necessary to engage more directly with norms and attitudes around violence to achieve a shift away from violence. Alternatively, it is possible that the intervention period was simply not long enough to allow for the complete replacement of violent discipline in favour of these new methods and that a longer and more sustained engagement with teachers would have ultimately shifted behaviours away from corporal discipline.

The intervention activities and reflection exercises were supposed to improve teacher attitudes and self-control by strengthening their skills and competencies; however, our results showed that there were no differences in levels of empathy, growth mindset and self-efficacy between intervention and control teachers. It is therefore possible that the intervention content led to the small shifts in attitudes and self-regulation that were observed at midline and endline directly. Finally, improved social support was supposed to sustain and facilitate this change process but again we found no difference in the level of support reported by teachers in intervention and control schools; we conclude therefore that teachers could benefit from more formal and continued coaching and mentoring during the programme period.

### Strengths and limitations

Our study had several strengths and some limitations. Strengths include high student and staff response rates and triangulation of a wide range of data including evaluation data collected as part of a randomised controlled trial, routine monitoring data collected by the International Rescue Committee, and attendance records filled out by teachers themselves during intervention sessions. Even though the process evaluation was designed prospectively and fully embedded into the overall study design our ability to make linkages between routine monitoring data and evaluation data was limited. Furthermore, the process evaluation analyses took place after findings from the impact evaluation were known which required changes to our analytical approach. Some of the analyses presented wide confidence intervals making it difficult to rule out moderate associations. As expected, some of the routinely collected attendance and homework completion data were incomplete, and process measures collected as part of programme implementation had not been tested to determine validity and reliability. Additionally, self-reported survey data and data from classroom observations may have been affected by social desirability bias.

### What works (and what doesn’t) to prevent violence against children in schools?

We contribute to a limited, but growing, evidence base on strategies to address teacher violence. Of the handful of robustly evaluated interventions globally that achieved reductions in levels of teacher violence, very limited scrutiny of implementation strategies and mechanisms of action has been undertaken. This leaves open questions about how and which components of these complex interventions led to changes in behaviours.

Violence prevention is often addressed through complex multi component interventions characterised by long causal chains [17, 18, 20]. Understanding how these programmes work requires carefully designed evaluations that assess change along the pathways and enable identification of key mechanisms. When interventions fail to achieve their expected outcomes, it’s important to understand whether the programme theory was incorrectly formulated or whether implementation or contextual factors harmed a potentially successful intervention. A process evaluation of the Good School Toolkit showed that reductions in violence in Ugandan primary schools were achieved through encouragement and reward of positive student behaviours and reinforcement of teachers’ positive discipline methods suggesting that a key component of the intervention was the support provided to individuals to learn new ideas and behaviours that generated new norms supportive of positive student-teacher relationships [37]. Evidence showed that the Toolkit triggered change across several dimensions including students’ feeling of safety and connectedness, school governance structures, teacher-student and peer-to-peer relationships, in addition to teaching and disciplinary approaches [37, 38]. Limited evidence is available on the mechanisms that led Right to Play’s intervention in Pakistan, which used an intense life-skills and play-based curriculum for students complemented by training for parents and school staff, to reductions in teachers’ use of corporal punishment [18]. However, it was suggested that the intervention empowered children by improving their communication and conflict-resolution skills and through changes in social norms around violence and interpersonal relations [18, 39]. Shifts in social norms and attitudes, combined with improved communication and of conflict resolution skills were also the hypothesised kay pathways to lower levels of violence for a school based intervention implemented in Afghanistan [20]. The Irie Classroom Toolbox instead was hypothesised to have achieved reductions in violence by improving teachers’ wellbeing and self-efficacy and promoting their use of appropriate teaching techniques [19, 21]. Despite the fact that the intervention did not explicit discourage use of violence, it offered teachers tools to promote the creation of a safe and nurturing classroom environment [21]. Although it is less clear which specific aspects of the intervention were responsible for these changes, evidence suggests that its main mechanisms of action were indeed linked to improvements in teacher wellbeing and skills.

EmpaTeach’ s inability to achieve the anticipated reductions in physical and emotional violence from teachers generated an opportunity to reflect on what intervention components may be necessary or essential. EmpaTeach included several elements that were similar to those of successful interventions such as quality content on alternative classroom management and disciplinary strategies and the use of a group setting for intervention delivery that promoted social support. However, the uptake of such strategies alone was not sufficient to shift teachers’ behaviours away from violent discipline. The social dynamic provided by the peer-groups did not provide teachers with the required support to allow for these new practices to consolidate.

Results from our analyses and other studies seem to suggest that successful prevention of teacher violence is more likely in the context of whole-school interventions that involve a variety of stakeholders within schools and communities, foster changes in the overall school environment, focus on promoting alternative disciplinary methods for teachers, and offer continued mentoring and support throughout the complex process of change [5, 20, 37]. Despite differences in intervention content and delivery, most interventions achieved reductions in teacher violence through improvements in teacher-student relationships. In EmpaTeach, although empathy and perspective-taking abilities were used as key levers for change, teachers’ relationship with students was not the main focus of the intervention.

Evidence also shows that a prolonged engagement with school actors may be important to support complex changes in attitudes and behaviours around violence, but the delivery of long interventions is challenging in rapidly-changing fragile settings. Identifying essential intervention ingredients is a pressing need to prevent and reduce violence in humanitarian contexts in an efficient and effective manner.

Based on our results, we would suggest further exploration of three intervention components: 1) the current content on alternative discipline practices could be disseminated among other school stakeholders, beyond class teachers, to promote changes in the overall school climate and create more social support and championing; 2) ways to engage with norms around violence more directly could be explored, e.g. respected members of the community or individuals with high credibility among the teacher body could be trained to facilitate critical reflection on norms and explicitly discourage use of violence;3) coaching and mentoring for teachers and in-classroom feedback and support could be integrated to strengthen skills acquisition. Generally, further testing of light-touch programmes in a variety of settings is advisable to pinpoint which intervention components produce desired changes in which contexts.

## Conclusion

The findings of these secondary analyses of the PVACS trial offer some important insights into why the EmpaTeach intervention did not lower levels of violence from teachers to students in Nyarugusu Refugee Camp and highlight the importance of conducting theory-informed process evaluations alongside randomised trials to generate evidence on the role of the different elements of complex school-based interventions and interrogate mechanisms of impact. We encourage academics and practitioners working on promising violence prevention interventions to embed, connect or merge adaptable process evaluations into their evaluation designs [40] and explore how programmes could be compressed to be adapted and tested in emergency contexts. With new humanitarian crises unfolding every day finding effective ways to provide safe education environments for displaced and refugee children is critical.

## Supporting information

S1 Text Classification of teacher responses to PVACS survey questions about classroom scenarios. (DOCX)

S1 Table Intervention effect on teacher self-reported use of strategies at midline. (XLSX)

S2 Table Association between school mean level of teachers’ use of positive strategies and student self-reported experiences of violence at midline. (XLSX)
