# Supplementary material for: Understanding why EmpaTeach did not reduce teachers’ use of violence in Nyarugusu Refugee Camp: A quantitative process evaluation of a school-based violence prevention intervention
Source: PLOS Glob Public Health. 2023 Jun 14;3(6):e0001404. doi: 10.1371/journal.pgph.0001404 (PMC10266646; doi:10.1371/journal.pgph.0001404)
Supplement: S1 Text — (DOCX) [file pgph.0001404.s001.docx]

S1 Text. Classification of teacher responses to PVACS survey questions about classroom scenarios

| Multiple choice survey question | Responses coded as positive practices |
| --- | --- |
| 524. Can you tell me what you usually do when you get upset or annoyed in class to try to relax or regain focus? | The teacher takes a walk for a few minutes  The teacher prays  The teacher tries to control his/her breathing  The teacher counts to 10 to relax or takes deep breaths  The teacher takes a polite pose |
| 525. Can you tell me what happens most of the time in your class when one or more students are talking to each other and being distracted while you are explaining/teaching to the class? | The teacher stops talking and stares at the misbehaving students  The teacher asks students to sit properly for 1 minute  The teacher lowers his/her voice  The teacher claps his/her hands to regain students’ attention  The teacher explains that disturbing is wrong and/or asks them to stop  The teacher moves students’ seats around to separate the disturbing students  The teacher moves closer to the students who are distracting  The teacher takes away the item that created distraction |
| 526. Can you tell me what do you usually do when one or more students arrive late to class? | The teacher asks the student to apologise for the delay to the teacher or to the other students  The teacher makes the student stay longer after class or during break as punishment |
| 527. Can you tell me what do you usually do when one or more students perform well in class? For example, when a student gives the right answer to a question, or when a student helps other classmates with learning/assignments, or when students perform well on an assignment. | The teacher asks other students to clap or cheer for the student who performed well  The teacher praises the student  The teacher recognizes the students in front of the classroom  The teacher and classmates sing a song for the good student  The teacher gives the student more work/homework for fun or to help the student learn more (not as a punishment)  The teacher gives the student a prize  The teacher uses the student as a role model  The teacher provides advice to continue doing well  The teacher asks the student to help his peers at home  Ask the student to sit close to those who are doing poorly |
